# CRISPR deletion of *MIEN1* in breast cancer cells

**DOI:** 10.1371/journal.pone.0204976

**Published:** 2018-10-04

**Authors:** Timothy Van Treuren, Jamboor K. Vishwanatha

**Affiliations:** 1 Department of Microbiology, Immunology and Genetics, Institute for Cancer Research, University of North Texas Health Science Center, Fort Worth, Texas, United States of America; 2 Texas Center for Health Disparities, University of North Texas Health Science Center, Fort Worth, Texas, United States of America; University of Alabama at Birmingham, UNITED STATES

## Abstract

Migration and Invasion Enhancer (MIEN1) is an oncogene which is involved in facilitating motility of cancer cells through actin dynamics and gene expression. Increased MIEN1 expression in many types of tumors leads to disease progression and metastatic propensity. It is unclear precisely how MIEN1 is involved in this process and more studies are required to tease out the mechanisms. Here we show that Clustered Regularly Interspaced Short Palindromic Repeat (CRISPR) genome editing effectively produced specific genomic deletions in the *MIEN1* gene which led to the abrogation of its expression in breast cancer cells. The single guide RNAs (sgRNAs) mediated targeting of *MIEN1* was specific and none of the clones screened for off-target cleavage revealed any insertions or deletions (indels). Additionally, disruption of the *MIEN1* gene did not alter the cell morphology, growth, proliferation or survival. Knocking out *MIEN1* in these breast cancer cells will allow future studies to determine the exact role MIEN1 plays in breast tumor metastasis, which might lead to production of novel therapeutics to treat this and other cancers.

## Introduction

**Fig 1:**
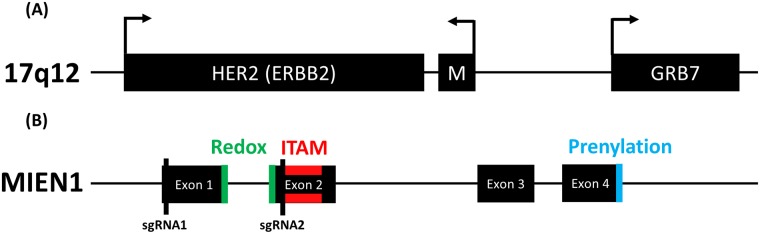
*MIEN1* gene location and structure. (A) Chromosome 17q12 genomic locus/*HER2* amplicon. Genomic location and orientation of *MIEN1* gene (M) in relation to *HER2* and *GRB7* within the *HER2* amplicon. (B) Structure of *MIEN1* gene. Exons of *MIEN1* with locations of the redox, immunoreceptor tyrosine activation motif (ITAM) and prenylation domains. Location of sgRNA sequences are denoted by vertical black lines.

*Migration and Invasion Enhancer I* (*MIEN1*) is a gene that is located on the long arm of the human chromosome 17, 0.5kb away from the *ERBB2* gene which encodes for the epidermal growth factor receptor Her2 (Fig 1A) [1]. Due to its proximity to the *ERBB2* gene, *MIEN1* is commonly amplified in breast cancer along with *ERBB2* which leads to an overexpression of MIEN1 protein in many breast cancers [1]. MIEN1 has been shown to functionally increase the invasive and migratory phenotype of various types of cells including breast cancer, prostate cancer, oral cancer, fibroblasts and endothelial cells [2–5]. MIEN1 increases expression of proteins known to be involved in metastatic processes such as matrix metalloproteinase 9 (MMP9) and vascular endothelial growth factor (VEGF) through activation of protein kinase B (Akt) and spleen tyrosine kinase (Syk) [2, 5, 6]. Actin cytoskeletal dynamics, an important component of cellular locomotion, are also regulated by MIEN1 acting through cofilin and focal adhesion kinase (FAK), particularly in the leading edge of the cell in the lamella [7]. These data indicate that MIEN1 is an important molecule which sits at the crossroads of cytoskeletal dynamics and signaling cascades which culminate in metastatic function and gene expression. It is important to understand the context of the role of MIEN1 in increasing cell motility and aggression in order to estimate its use as a prognostic biomarker and therapeutic target in the future.

Clustered Regularly Interspaced Short Palindromic Repeat (CRISPR) based genome editing has been at the forefront of molecular biology in the last several years. This technique promises quick, efficient genome modification. The modified cells that result from CRISPR genome editing can then be used to study the effect long-term loss of a specific gene has on signaling events and cellular function. Studying the roll of MIEN1 in cancer progression can be accomplished by a variety of methods; however, genomic deletion using the CRISPR-Cas9 system offers the ability to examine the functional significance of MIEN1 knockout (MKO) in a background in which it is endogenously expressed.

## Materials and methods

### Cell lines and culture conditions

The human epithelial breast cancer cell line MDA-MB-231 was obtained from the American Type Culture Collection (Manassas, VA, USA). The MDA-MB-231 derived organotropic metastatic variants 831 (brain) [8], 1833 (bone) [9], and 4175 (lung) [10] were provided as gift from Dr. Joan Massagué, Memorial Sloan Kettering Cancer Center (New York City, NY, USA). Before shipment, the cell lines were authenticated by STR analysis with the Promega PowerPlex Fusion V1.0. All three cell lines tested negative for mycoplasma infection when tested with MycoAlert PLUS from Lonza (Basel, Switzerland). The cell lines were confirmed to be mycoplasma free prior to use. All cell lines were cultured in DMEM high-glucose (HyClone) supplemented with 10% FBS, 4.05mM glutamine, 100IU penicillin, 100IU streptomycin and 0.25ug/ml Amphotericin B. Cultures were maintained in a humidified incubator at 37°C with 5% CO_2_.

### SgRNAs, plasmids and cloning

In order to efficiently target the *MIEN1* gene for KO, CRISPR guide sequences were selected using Benchling biology software based on the following criteria: the single-guide RNA (sgRNA) sequence should A) have a high on-target score [11], B) have a high specificity score (low off-target effects) [12] C) be found within an exon of *MIEN1*, D) be found within an exon specific to *MIEN1*, E) be found within an exon integral to the function of MIEN1, F) be located close to the N-terminus. sgRNA-A was chosen for its proximity to the translation start site while sgRNA-B was chosen for its location within an exon containing two of the three functional domains of MIEN1 (Fig 1B). Sequences for sgRNA oligos can be found in S1 Table.

sgRNA oligos were cloned into vectors according to the provided protocols from Addgene. The pSpCas9(BB)-2A-GFP (PX458) plasmid, which contains both Cas9 endonuclease/EGFP and sgRNA expression cassettes, was a gift from Feng Zhang (Addgene plasmid # 48138) [13]. The MLM3636 plasmid, which contains an sgRNA expression cassette, was a gift from Keith Joung (Addgene plasmid # 43860).

### Transfection and CRISPR genome editing

Cells were co-transfected with PX458 and MLM3636 plasmids using jetPRIME (VWR) as recommended by the manufacturer. 24-hours post transfection, GFP positive cells were FACS sorted on a Sony SH800 Cell Sorter. Single cell clones were grown and screened via PCR and immunoblotting for MIEN1 KO.

### Sequencing

Regions surrounding sgRNA target/off-target sites within the *MIEN1*, *LRP8* and *SQSTM1* genes were amplified by PCR using Amplitaq Gold 360 PCR Master Mix (Invitrogen). PCR reactions were cleaned using a GeneJET PCR Purification Kit (Thermo Scientific). Amplicons were then analyzed by Sanger sequencing (ACGT, Inc.).

### Western blot and antibodies

Proteins were harvested by lysing cells with RIPA lysis buffer (150mM NaCl, 1% NP-40, 0.5% Na deoxycholate, 0.1% SDS, 50mM Tris) supplemented with Protease Inhibitor Cocktail Set I (Millipore). Cell lysates were sonicated and centrifuged for 15 minutes at 4800 rpm at 4°C. Protein concentrations were measured using a Pierce BCA Protein Assay Kit (Thermo Scientific). Cell extracts were separated using a 4–12% Bis-Tris NuPAGE gel (Life Technologies Corporation) before being transferred to a nitrocellulose membrane (iBlot, Invitogen). Membranes were blocked with 5% Bovine Serum Albumin (BSA) (Sigma) in Tris-buffered saline containing 0.05% Tween 20 (TBST) at room temperature for 1 hour. Membranes were then incubated with agitation either 2.5 hours at room temperature or overnight at 4°C with primary antibody in 2.5% BSA in TBST. Following four 5 minute washes, membranes were then incubated with the appropriate HRP conjugated secondary antibody (SouthernBiotech) in 5% milk in TBST at room temperature for 1 hour before being washed four times for 5 minutes each. Finally membranes were developed using Immobilon Western Chemiluminescent HRP substrate (Millipore). Images were captured using and Alpha-imager Fluoretech HD2. Antibodies: 1) mouse anti-MIEN1 MO2 monoclonal raised against full-length recombinant MIEN1 (Abnova. 1:1000 dilution), 2) mouse Guide-it Cas9 polyclonal raised against full-length Cas9 from *Streptococcus pyogenes* (Clonetech, 1:1000 dilution), 3) mouse anti-β-actin C4 monoclonal raised against chicken actin (Santa Cruz Biotechnology. Antibody registry # AB_2714189. 1:2000 dilution).

### Morphology

Cells were grown on treated cell culture dishes and pictures were captured on an inverted light microscope using an attached Canon digital camera.

### Cell proliferation

5.0x10^3^ cells were plated into each well of a 96-well plate with 200ul of complete medium. Every 24 hours, 10ul of 10.5mg/ml 3-(4,5-dimethylthiazol-2-yl)-2,5-diphenyltetrazolium bromide (MTT) was added to 4–8 of the wells. Cells were incubated with MTT at 37°C for 2 hours to allow formation of formazan. Media was carefully removed and 150ul of DMSO was added to each well. Wells were mixed well to ensure formazan crystals were solubilized. Absorbance was read at 570nm. DMSO blank was subtracted from the absorbance of each sample. To measure the initial time point (T0), MTT was added to cells 2 hours after seeding experiment to allow for cell attachment. Media in other wells was changed every 48 hours. Results represent five independent experiments performed in quadruplicate.

### Cell survival

2.0x10^4^ cells were plated into each well of a 96-well plate with 200ul of complete medium. The next day, media was changed to serum free medium. MTT assay was then performed as outlined above. Results represent five independent experiments performed in quadruplicate.

### Spheroid growth

1.0x10^3^ cells in 50ul were plated into each well of a low-attachment U-bottom 96-well plate. The plate was briefly centrifuged to collect cells at the bottom of the well. With the plate on ice, 50ul of 5% Matrigel (Corning) was added to each well. The plate was again spun. The plate was then placed at 37°C for 30 minutes before 100ul of complete media was added to the top of the solidified Matrigel. Spheroids formed overnight and images were collected on an inverted light microscope every 24 hours as outlined above. Total area of spheroids was measured using the “Analyze Spheroid Cell Invasion In 3D Matrix” macro in ImageJ. Data was taken from four independent experiments performed in triplicate.

### Statistical analysis

The Student t-test was used for statistical analysis. Data are presented as mean ± standard error of the mean. * denotes a p-value of less than 0.05 and was considered to be significant. *** denotes a p-value of less than 0.005.

## Results

### *MIEN1* deletion and PCR screening

**Fig 2:**
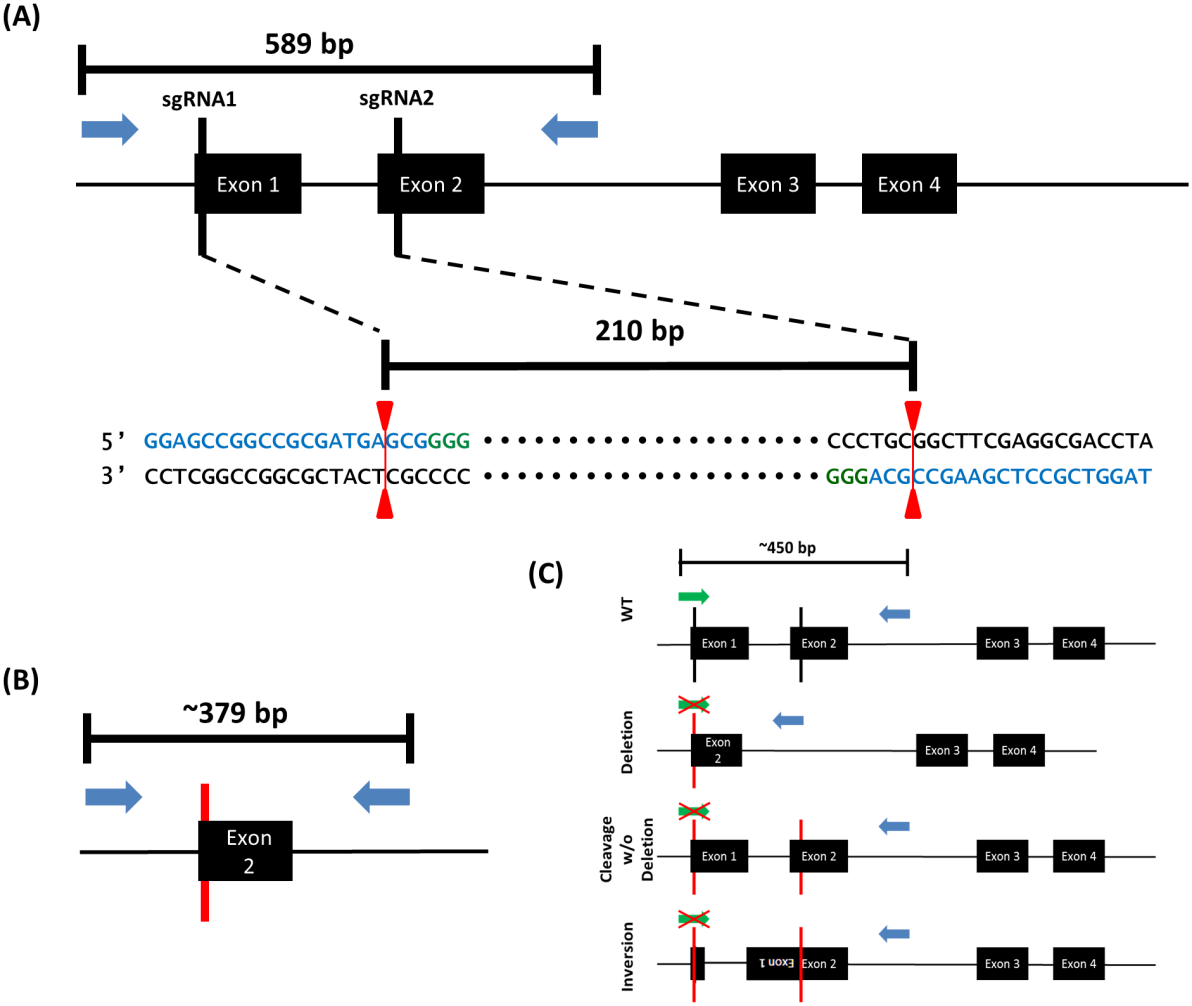
*MIEN1* Deletion and Indel PCR reactions. (A) *MIEN1* deletion schematic. Blue arrows represent location of primers for deletion PCR reaction. Blue sequences are sgRNA sequences. Green sequences are PAM sequences. Red line indicates approximate cleavage location to yield 210bp deletion. (B) Genomic deletion of *MIEN1* exon 1. PCR product result following deletion of sequence between sgRNA1 and sgRNA2. (C) Indel PCR diagram. CRISPR cleavage scenarios other than deletion are outlined. The PCR primer which lies across the sgRNA1 target site is sensitive to indels which occur during cleavage without deletion or inversion. Absence of band in indel reaction indicates presence of indel. Green arrow indicates sgRNA1 Oligo1 which acts as the forward primer. Blue arrow is the same reverse primer from the deletion reaction. Red vertical lines are indels. Red ‘X’ indicates an inability for the forward primer to anneal.

**Fig 3:**
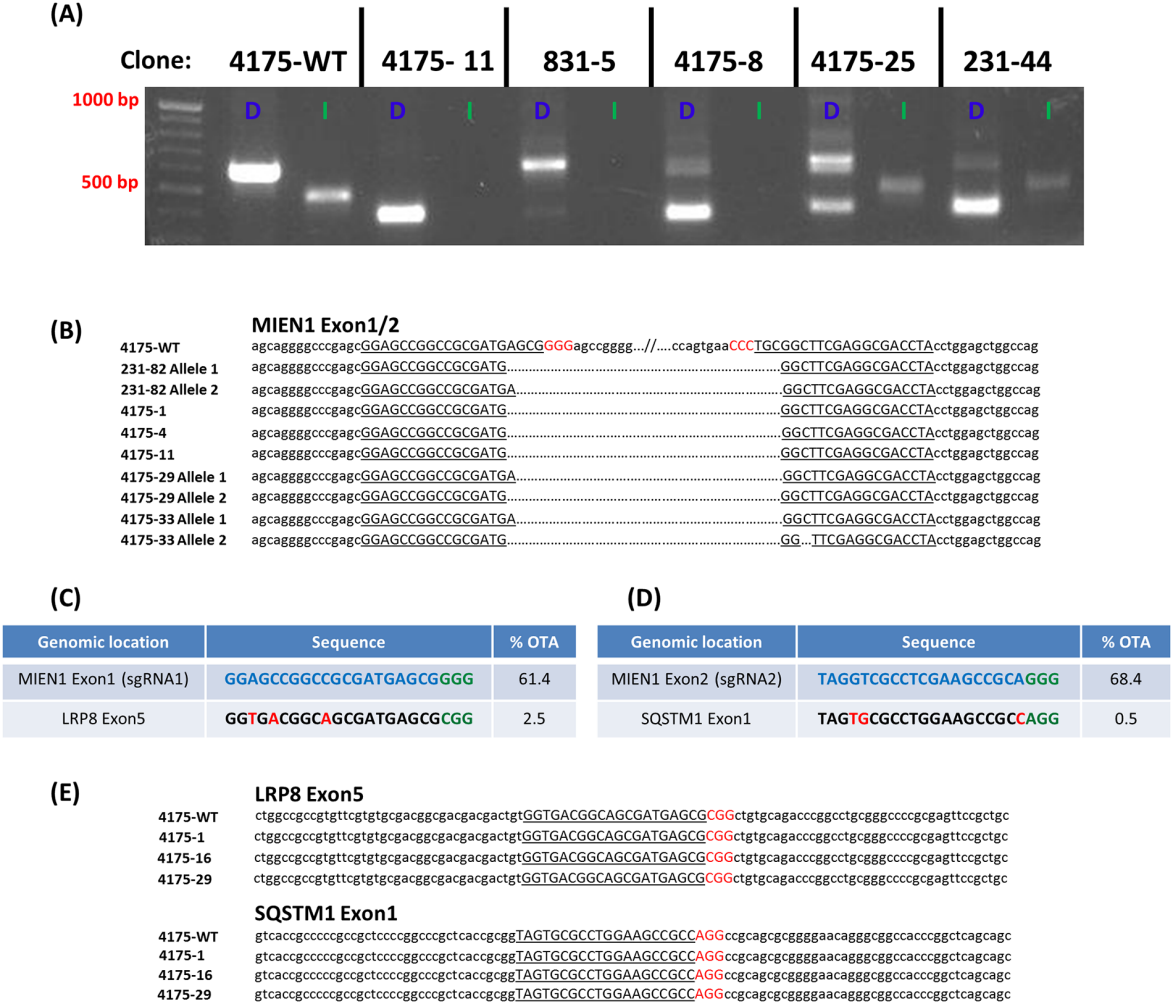
PCR screening and sequencing. (A) Deletion and indel PCR screening. Representative clones exhibiting all possible band combinations. WT: wild-type. D: Deletion. I: Indel. (B) *MIEN1* deletion sequences. Representative sequences of several clones which showed the smaller band in the deletion PCR reaction resulting from deletion and NHEJ. Underlined sequences correspond to remains of sgRNA target sequences. Red sequences correspond to PAM sequences. (C and D) sgRNA off-target. sgRNA sequences with on-target activity score (0–100, higher is better). Second line is sequence of highest scoring off-target location within a gene. Red letters denote mismatches from the *MIEN1* targeting sgRNA sequence. (E) Off-target sequencing. Representative sequences of several clones which show no indels at the off-target loci.

To screen FACS sorted single cell clones, a deletion strategy using two sgRNA sequences was used to enable rapid, high-throughput analysis of genomic alterations [14]. Co-transfection of the two sgRNAs allows for simultaneous Cas9 endonuclease-mediated cleavage at both target sites. During the non-homologous end joining (NHEJ) repair process following simultaneous cleavage, the intervening DNA sequence is lost. It has been shown that NHEJ after deletion using two sgRNAs often results in precise repair without large insertions/deletions (indels) [15]. Therefore, the deletion of DNA can be easily resolved on an agarose gel following PCR amplification across the deleted segment. Primers were designed flanking the two sgRNAs targeting the *MIEN1* gene which yielded a 589bp amplicon (Fig 2A). Following deletion and NHEJ, the deletion amplicon is reduced to approximately 379bp (Fig 2B). In addition to simultaneous cleavage, deletion and NHEJ, there are several other possible Cas9 cleavage mechanisms (Fig 2C). First, simultaneous cleavage occurs, but the intervening segment in not deleted and is instead re-inserted during NHEJ, which introduces indels at both target sites. Second, cleavage occurs at one of the target sites and NHEJ is performed before cleavage occurs at the other target site. Third, simultaneous cleavage occurs, however the intervening segment is inverted and then re-inserted during NHEJ. Fourth, cleavage and NHEJ occurs at only one of the two sites. All of these scenarios should also result in gene product KO, but none of them are able to be detected using the deletion PCR reaction alone as they will all most likely yield an amplicon close to the wild-type length. In order to be able to identify clones which were edited by these alternate mechanisms, we designed an indel PCR reaction using one of the sgRNA oligos which was cloned into the PX458 vector (S1 Table). Since this oligo contained the sgRNA sequence, it straddled the Cas9 cleavage site and its annealing was sensitive to indels (Fig 2C). Single cell clones were then screened by both the deletion and indel PCR reactions to identify potential MIEN1 KO clones (Fig 3A). 47% (72/151) of clones which were screened by PCR exhibited genomic lesions either by presence of the smaller band in the deletion reaction or absence of a band in the indel reaction. These clones were selected as candidates for western blot screening.

### On-target and off-target sequencing

Several clones which were chosen for western blotting were sequenced to examine the genomic lesion at the *MIEN1* locus following deletion as well as potential off-target cleavage. *MIEN1* deletion bands were PCR amplified and sequenced (Fig 3B). Several of the deletion bands showed perfect deletion and repair without any indels. There were also several single nucleotide deletions of the adenine immediately upstream of the sgRNA1 cut site. This validated the efficient NHEJ that allowed us to be able to determine the deletion of a segment of the *MIEN1* gene by PCR. The sgRNA sequences which were used to target the *MIEN1* gene both had high overall off-target scores, indicating low probability of off-target effects. To examine potential off-target CRISPR-mediated cleavage and indel formation, the highest scoring off-target locus within a gene for each sgRNA was sequenced (Fig 3C–3E). All 19 *MIEN1* deletion clones which were sequenced at these potential off-target loci showed no indels at either the *LRP8* or *SQSTM1* locus, indicating no off-target effects (Fig 3E).

### MIEN1 knockout screening

**Fig 4:**
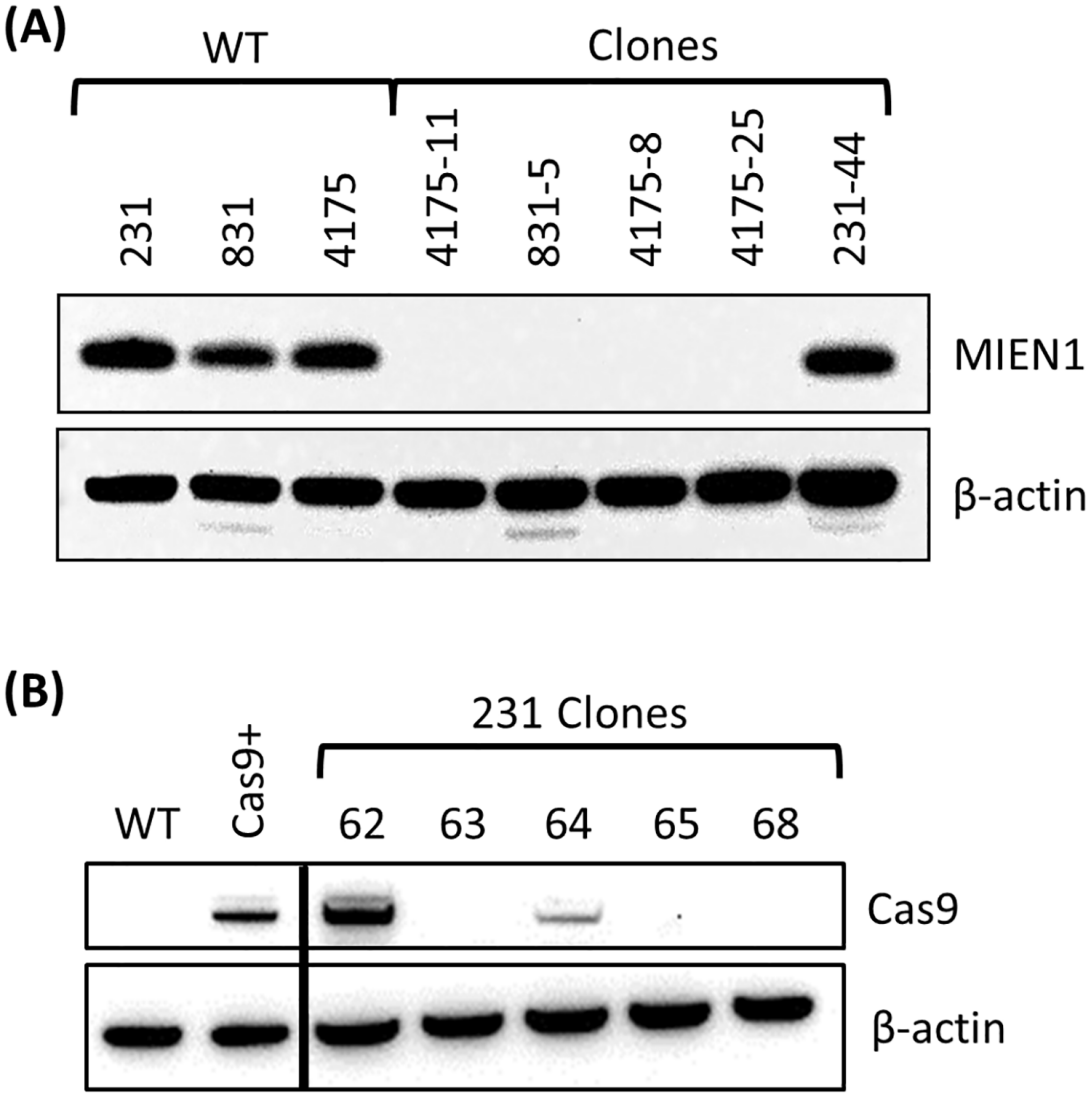
MIEN1 KO western blot. (A) MIEN1 western blot. Parental wild-type (WT) cell lines with clones which match the PCR reactions from Fig 3A. (B) Cas9 western blot. Representative western blot from Cas9 screening of MIEN1 KO clones. Cas9+ sample was pool of cells transfected and selected with Cas9 expressing plasmid. Original, full-length blots can be found in S1 Fig.

Clones selected based on deletion and indel PCR reactions were screened by western blot for MIEN1 knockout (KO) (Fig 4A). 85% (50/59) of the clones which were selected from PCR screening were negative for MIEN1 protein. Some clones which amplified both small and large bands during PCR, clone 231–44 for instance (Fig 3A), still showed MIEN1 expression following western blot, indicating that some clones retained one unmodified allele following CRISPR editing. Additionally, it is important to ensure that KO clones are Cas9-negative to reduce the potential for off-target cleavage, which would be the case if the nuclease was stably expressed through genomic insertion. Only 16% (8/50) of those clones which were MIEN1 negative expressed Cas9. These clones were not used in subsequent experiments.

### Effect of MIEN1 KO on morphology, growth and survival of breast cancer cells

**Fig 5:**
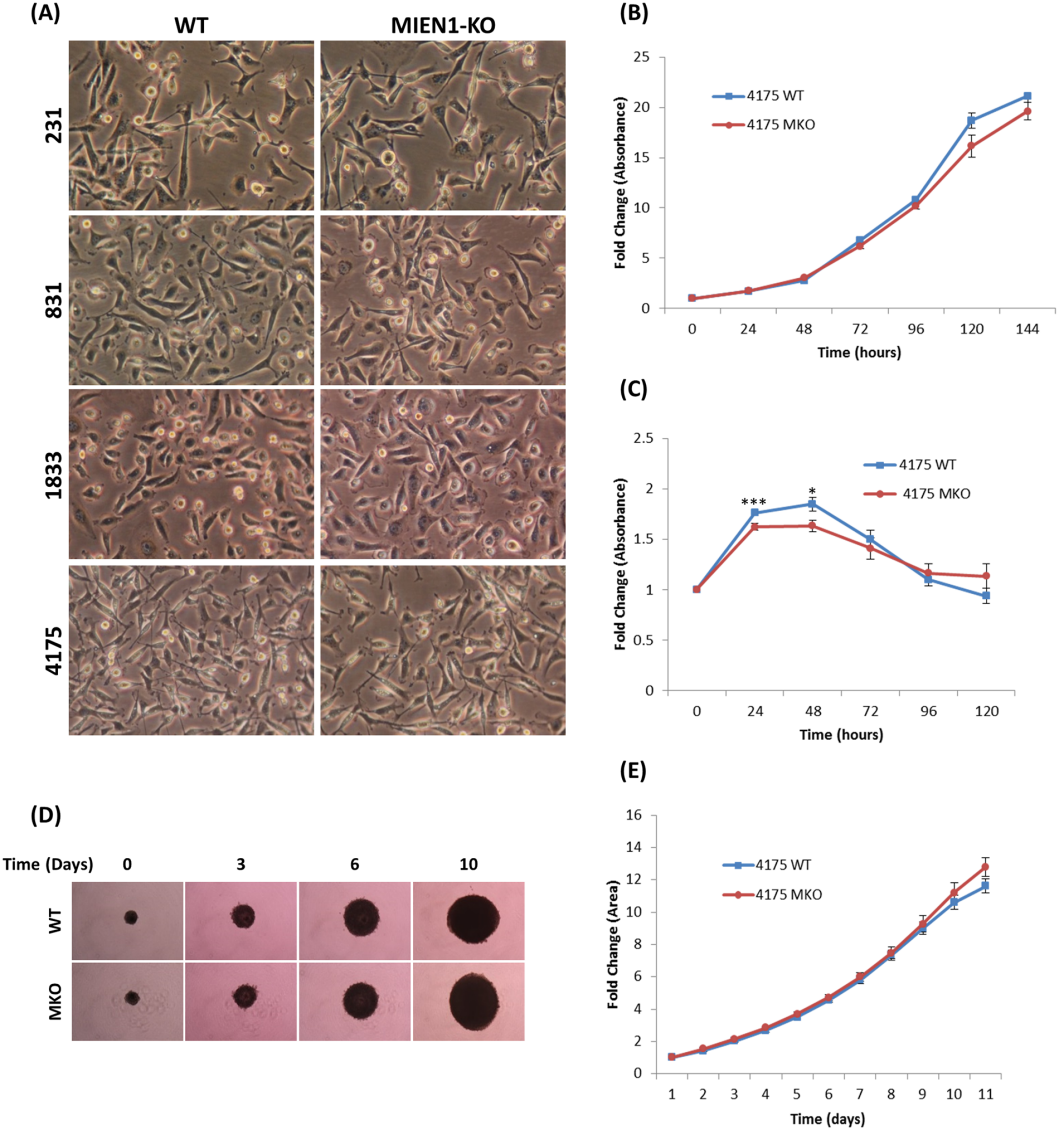
MIEN1 knockout cell characterization. (A) Cell morphology. Representative 10x magnification phase contrast images of MIEN1 KO cells and corresponding parental cells. (B) MTT assay comparing fold change of proliferation of 4175-WT and 4175-MKO cells. (C) MTT assay comparing fold change of cell survival during serum deprivation of 4175-WT and 4175-MKO cells. (*p-value <0.05. ***p-value <0.005) (D) Spheroid formation. Representative 4x magnification phase contrast images of spheroid growth in Matrigel for 4175-WT and 4175-MKO cells. (E) Quantification of fold change of area of spheroids. Error bars are standard error.

MIEN1 KO cell lines were produced by pooling all clones which showed no MIEN1 expression following western blotting. In order to see the effect of MIEN1 KO on general cellular characteristics, we first examined the morphology of the cells (Fig 5A). There was no observable difference in the morphology of the MIEN1 KO cell lines when compared to their parental cell lines. Next, we performed MTT assays using the 4175 lung metastatic cell line and its MIEN KO derivative to study proliferation (Fig 5B) and cell survival following serum deprivation (Fig 5C). There was no difference in the growth rate of the two cell lines as the 4175 cells had a doubling time of 32.7 hours while the 4175-MKO cells had a doubling time of 33.5 hours. Likewise, there was no observable difference in the time-course cell survival of the two cell lines. Several early time points did yield significantly different results between the two cell lines, however these differences were not consistent throughout the time-course. Finally, to see if MIEN1 KO had an effect on 3-dimentional spheroid growth, 4175 and 4175-MKO cells were grown in Matrigel (Fig 5D and 5E). Similar to the proliferation assay, no difference was seen in the growth of the cells in a more physiological, 3-dimentional environment. Together this data indicates that we have designed highly efficient sgRNAs that are able to efficiently and specifically target the *MIEN1* gene and that knocking out *MIEN1* does not affect the morphology, growth and survival of breast cancer cells.

## Discussion

CRISPR genome engineering has become a staple of molecular biology research over the last several years. There is still debate about the benefits and applications of CRISPR vs other gene expression regulation systems, such as RNA interference (RNAi). However, CRISPR has been shown to yield more reproducible data and minimize global off-target effects compared to RNAi [16, 17]. While there are concerns about the specificity and potential off-target effects of CRISPR-Cas systems [18, 19], much work is being done to improve the specificity of sgRNAs and reduce the off-target cleavage of Cas proteins through genetic mutation [11, 20, 21]. Here we have used the CRISPR-Cas9 system to eliminate MIEN1 protein from breast cancer cells which normally have high MIEN1 expression. Two sgRNAs were used to delete a segment from the *MIEN1* gene. Our results demonstrate that the co-expression of these sgRNAs with the Cas9 endonuclease resulted in a high on-target editing efficiency (47%) in four breast cancer cell lines with no off-target effects. The majority of these edited cells showed no MIEN1 protein expression when screened by western blot indicating that we have developed a potent system for producing MIEN1 KO cell lines for future experiments.

We and others have previously shown that MIEN1 is an oncogene involved in the migration, invasion and progression of breast, prostate and oral cancers and is associated with decreased overall survival [3–5, 22, 23]. MIEN1 mediates many pro-metastatic signals including activation of Akt kinase which facilitates downstream gene expression through NF-kB [3] and modulation of actin cytoskeleton to facilitate cell motility [22]. Because of the importance of MIEN1 in cancer progression and metastatic events, producing CRISPR mediated MIEN1 KO cells will allow us to better understand the role of MIEN1 in these various processes. Knocking out MIEN1 in these cancer cells allows us to study the effect of its absence from a background where it is normally expressed rather than over-expressing it in a cell line which does not have endogenous expression. We have confirmed through CRISPR deletion that MIEN1 does not play a role in the proliferation and survival of breast cancer cells, even within 3D culture. This platform will also allow us to further study how MIEN1 contributes to metastatic processes and validate its potential as a therapeutic target and prognostic biomarker.

## Supporting information

S1 Fig Full western blots from Fig 4. (TIF)

S1 Table Oligos. Sequences and orientations of oligos used within this study. Primers used for sequencing are denoted with *.
(XLSX)
