# Supplementary figures and images for: CRISPR deletion of MIEN1 in breast cancer cells
Source: PLoS One. 2018 Oct 4;13(10):e0204976. doi: 10.1371/journal.pone.0204976 (PMC6171864; doi:10.1371/journal.pone.0204976)

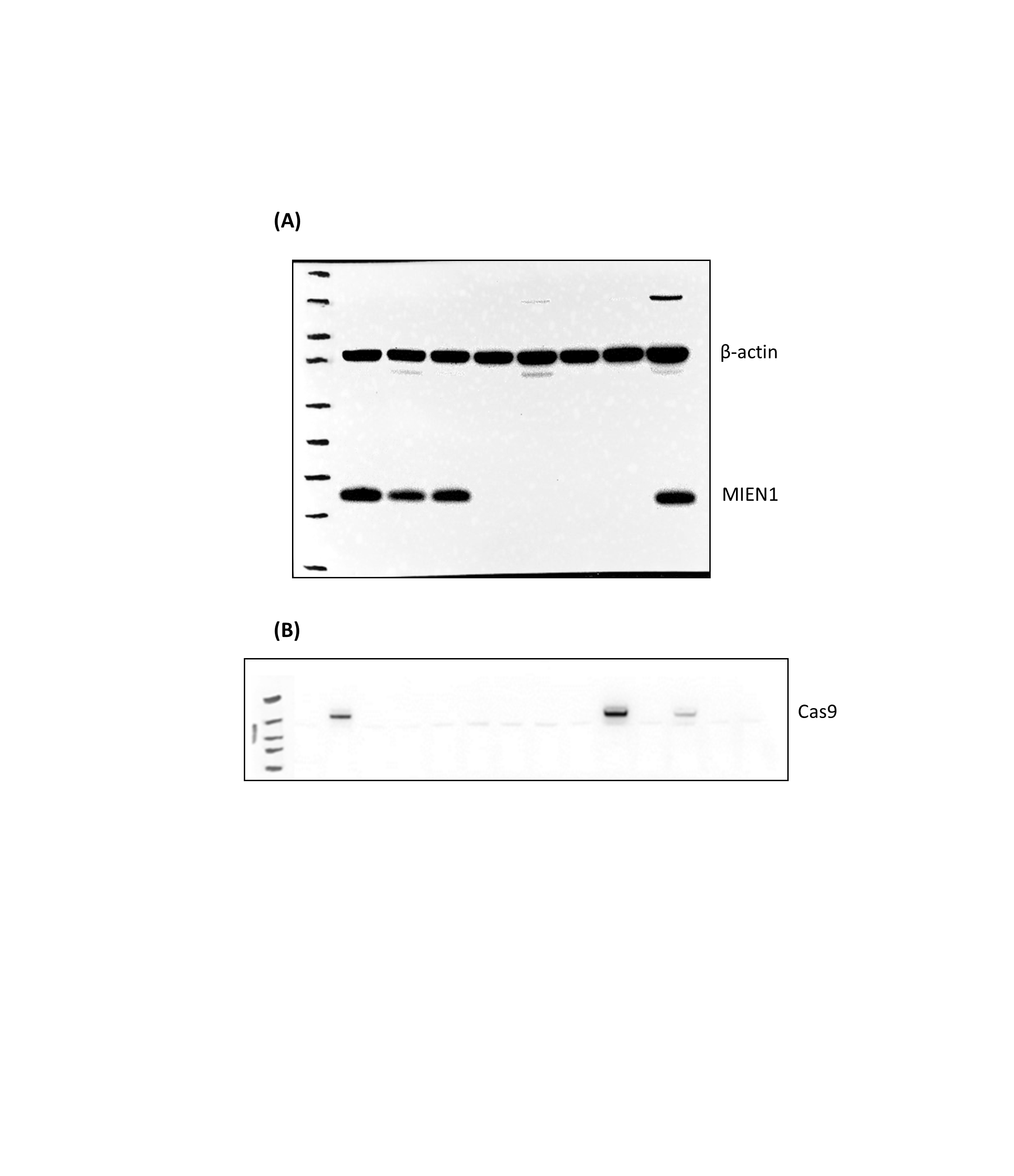

Supplement: S1 Fig — (TIF) [file pone.0204976.s001.tif]
